# Entrapment of intravitreal triamcinolone behind the crystalline lens

**DOI:** 10.4103/0301-4738.53066

**Published:** 2009

**Authors:** Amjad Salman, Pragya Parmar, Vanila G Coimbatore, Rajmohan Meenakshisunderam, Nelson Jesudasan A Christdas

**Affiliations:** Institute of Ophthalmology, Joseph Eye Hospital, Tiruchirapalli - 620 001, India

Dear Editor,

Intravitreal triamcinolone acetonide (IVTA) has been increasingly applied as a treatment of diabetic macular edema. Complications include glaucoma, cataract and endophthalmitis.[1–3] Complications such as endophthalmitis can produce a sudden, profound decrease in vision. We report a patient in whom triamcinolone became entrapped behind the crystalline lens following IVTA injection leading to a sudden but temporary visual loss.

A 49-year-old diabetic patient presented with proliferative diabetic retinopathy and macular edema in both eyes. Visual acuity was 20/60 in both eyes and the lens was clear. Fundus fluorescein angiography confirmed the presence of new vessels with diffuse leaks in the macula. The patient was treated with pan-retinal photocoagulation and grid laser. One month later, his visual acuity remained 20/60 in the right eye but dropped to 20/200 in the left eye. Optical coherence tomography (OCT) showed a diffuse thickening in the macula with a foveal thickness of 740 μ.

Under peribulbar anesthesia, 0.1 cc (4mg) of triamcinolone acetonide was injected in the inferotemporal quadrant 4mm from the limbus using a 26G needle in the left eye. At the moment of injection, the patient made a sudden jerky movement. After injection, the surgeon noted that the drug appeared immobile just behind the lens. As a part of routine protocol, the patient was instructed not to lie down for 6 hours.

**Figure 1:**
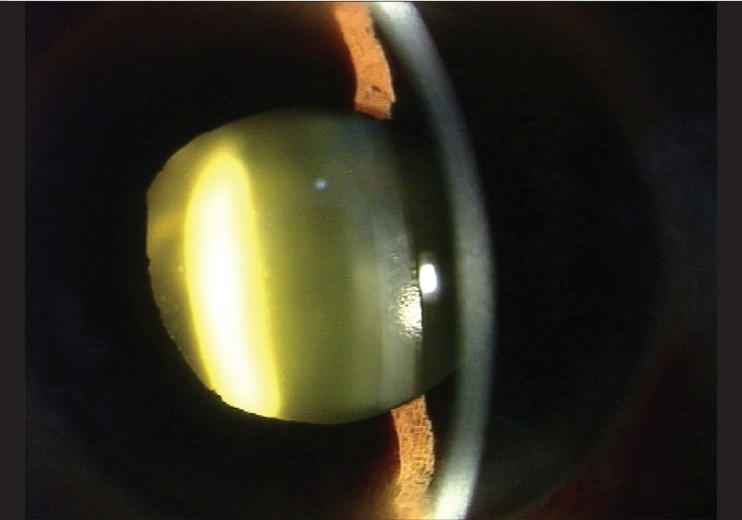
Appearance of the eye on the first day after intravitreal triamcinolone acetonide injection. The drug suspension is visible as a yellow deposit behind the crystalline lens

**Figure 2:**
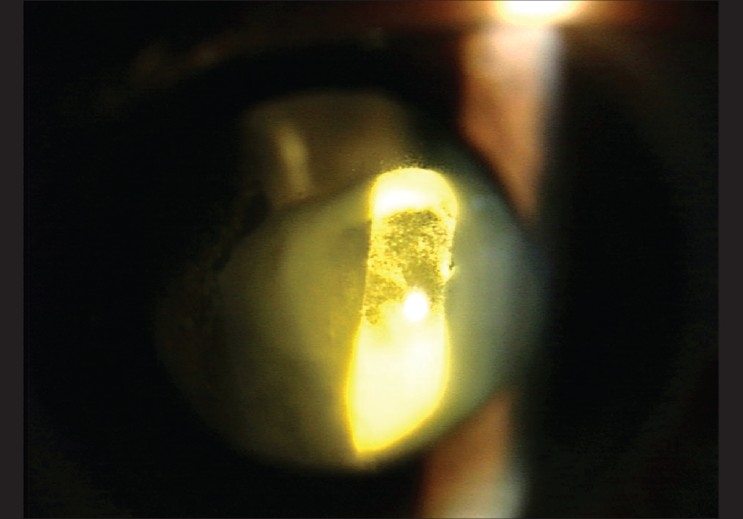
One week after the injection, the drug deposit has started to clear

**Figure 3:**
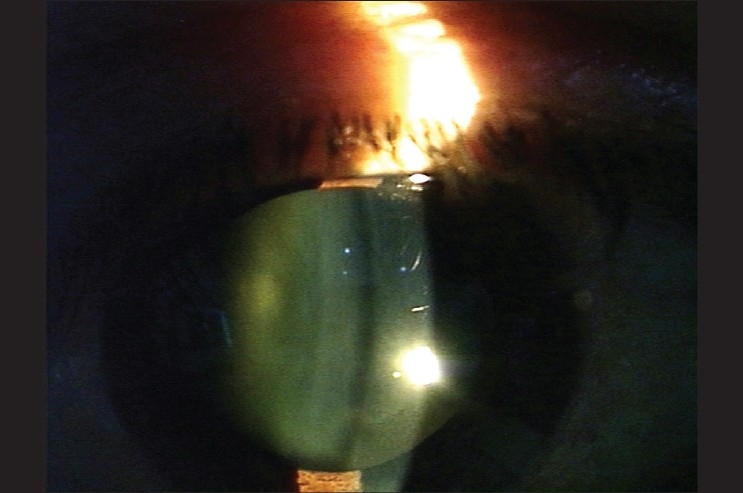
Two weeks after the injection, the drug has cleared completely while the lens remains clear

On the first post-operative day, the patient's visual acuity in the left eye dropped to hand movements. Slit lamp examination showed the drug between the posterior capsule of the lens and the anterior hyaloid face [Fig. 1]. There was no view of the fundus but an ultrasound B scan exam revealed a clear vitreous. One week later, the drug deposit started to clear [Fig. 2] and it cleared completely by two weeks leaving a clear lens [Fig. 3]. Visual acuity improved to 20/40 and the foveal thickness decreased to 260 μ. The lens remained clear with a follow up of 7 months.

Sudden decrease of visual acuity after IVTA injection is usually related to endophthalmitis. Anterior segment problems after IVTA are rare and include vitreous prolapse into anterior chamber with or without intraocular lens (IOL) decentration and opaque coating over the IOL.[4,5]

In our patient, we believe that the head movement at the time of injection caused the drug to be delivered into the Berger's space between the posterior lens capsule and the anterior hyaloid face where it remained trapped due to the normal adhesions. Although surgical removal via the pars plana was an option, we preferred a conservative approach to avoid damage to the lens. In any event, the drug cleared over the next 4 weeks with the lens remaining clear. This complication could have been probably avoided through better communication with the patient regarding the need to avoid movements during the procedure. This case highlights the importance of proper technique of intravitreal injections.

In conclusion, intravitreal triamcinolone acetonide may become entrapped behind the crystalline lens if the needle is not properly positioned. Conservative management allows the drug to clear without trauma to the crystalline lens.
